# Predictors of outcome of multidisciplinary treatment in chronic widespread pain: an observational study

**DOI:** 10.1186/1471-2474-14-133

**Published:** 2013-04-11

**Authors:** Aleid de Rooij, Marike van der Leeden, Leo D Roorda, Martijn PM Steultjens, Joost Dekker

**Affiliations:** 1 Amsterdam Rehabilitation Research Center | Reade, Jan van Breemenstraat 2, 1056AB, Amsterdam, The Netherlands; 2 Department of Rehabilitation Medicine and EMGO Institute, VU University Medical Centre, Amsterdam, The Netherlands; 3 School of Health, Glasgow Caledonian University, Glasgow,Scotland, UK; 4 Department of Psychiatry and EMGO Institute, VU University Medical Centre, Amsterdam, The Netherlands

**Keywords:** Chronic widespread pain, Fibromyalgia, Predictors, Multidisciplinary treatment, Outcome

## Abstract

**Background:**

The effectiveness of multidisciplinary treatment in chronic widespread pain (CWP) is limited. The considerable heterogeneity among patients is a likely explanation. Knowledge on predictors of the outcome of multidisciplinary treatment can help to optimize treatment effectiveness. The purpose of this study was to identify predictors of multidisciplinary treatment outcome in patients with CWP.

**Methods:**

Data were used from baseline and 6 months follow-up measurements of a prospective cohort study of 120 CWP. Regression models were used to assess whether baseline variables predicted treatment outcome. Outcome domains included: pain, pain interference, depression, and global perceived effect (GPE). Potential predictors included: psychological distress, illness and self-efficacy beliefs, fear-avoidance beliefs and behaviour, symptoms, disability, and socio-demographic factors.

**Results:**

Greater improvement in pain was predicted by more pain at baseline and male gender. Greater improvement in interference of pain in daily life was predicted by more interference of pain in daily life at baseline, lower levels of anxiety, a stronger belief in personal control, less belief in consequences, male gender, and a higher level of education. Greater improvement in depression was predicted by higher baseline values of depression, stronger beliefs in personal control, and a higher level of education. Better outcome on GPE was predicted by less pain, less fatigue, and a higher level of education.

**Conclusion:**

Less anxiety, stronger beliefs in personal control, less belief in consequences, less pain, less fatigue, higher level of education, and male gender are predictors of better outcome of multidisciplinary treatment in CWP. Tailoring treatment to these specific patient characteristics or selecting eligible patients for multidisciplinary treatment may further improve treatment outcome.

## Background

Chronic widespread pain (CWP) is a syndrome of unknown aetiology which is characterized by widespread musculoskeletal pain, fatigue and poor sleep [1,2]. A subcategory of patients with CWP also fulfil the criteria of fibromyalgia (FM) [3]. Chronic widespread pain and its associated symptoms result in a reduced quality of life and disability, and is associated with a negative long-term outcome [4].

Multidisciplinary treatment programs are recommended in patients with FM and CWP and the associated problems [5,6]. The multidisciplinary treatment is multimodal and often includes the efforts of a number of disciplines. Evidence for positive effects of multidisciplinary treatment in FM has been reported [7-9]. However, on average, the effects of multidisciplinary treatment are limited. A plausible explanation for this limited effect is the considerable heterogeneity among patients [7,10]. It is likely that the outcome of multidisciplinary treatment depends on the specific combination of symptoms, patient characteristics and treatment characteristics. Our systematic review revealed preliminary evidence for several patient characteristics and symptoms as predictors of the outcome of multidisciplinary treatment [11]. However, the evidence for these predictors is generally weak to inconclusive. Further studies are needed to establish the relationship between patients characteristics and the outcome of multidisciplinary treatment.

**Figure 1:**
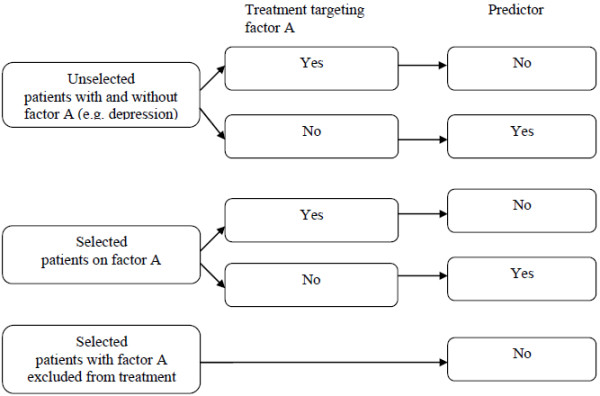
Selection of patients, nature of treatment and predictive value of patient characteristic.

Symptoms and patient characteristics may predict treatment outcome, depending on the selection of patients and the nature of treatment, as is illustrated in (Figure 1). Let us assume that factor A (e.g. depression [12]) predicts the persistence of symptoms and disability in untreated patients. In an *unselected* sample of patients and with treatment *not targeting* factor A, factor A is expected to predict a poor treatment outcome. Conversely, in an unselected sample and with treatment *targeting* factor A, factor A is not expected to predict treatment outcome: because factor A is targeted during treatment, it is expected to loose its predictive value. The same reasoning applies to a sample selected for factor A. Finally, in a *selected* sample, with patients with factor A excluded from treatment, factor A will not predict treatment outcome: if factor A is not present, it cannot predict treatment outcome.

Psychological distress (depression and anxiety) is commonly found in CWP patients [12,13] and is a prognostic factor for symptoms and disability [12,14]. Although patients with severe depression are typically excluded from multidisciplinary treatment, symptoms of depression and anxiety are common among CWP patients receiving multidisciplinary treatment. The focus of a multidisciplinary pain management program is often on cognitive and behavioural components of pain and disability, instead of depression and anxiety [6]. It is therefore expected that depression and anxiety predict a poor treatment outcome, as long as multidisciplinary treatment does not focus on the treatment of anxiety and depression.

A wide variety of cognitive concepts (e.g. illness beliefs, self-efficacy beliefs and fear-avoidance beliefs) have been shown to play an important role in chronic widespread pain (CWP). In our previous work we found overlap between these cognitive concepts [15]. Because of the explorative nature of this study we treated these concepts separately.

Illness and self-efficacy beliefs play an important role in the adaptation of the patient to symptoms of the illness and its associated problems [16-19]. Illness beliefs are ideas that patients hold about their illness [20,21]. Self-efficacy is defined as one’s confidence in performing a particular behaviour and overcoming barriers to that behaviour [22]. These beliefs are typically not a reason to reject patients for multidisciplinary treatment, nor is the altering of illness and self-efficacy beliefs structurally embedded in the multidisciplinary treatment in a standardized way. These factors are therefore expected to predict treatment outcome, as long as they are not the focus of treatment.

Fear-avoidance beliefs and behaviour (i.e. fear of pain, catastrophizing, and avoidance) are assumed to lead to persistence of pain and disability [23,24]. As multidisciplinary treatment aims at increasing activities in daily life despite of pain, fear avoidance beliefs and behaviour are often altered during multidisciplinary treatment [6]. Thus, although these factors are assumed to predict persistence of pain and disability, it is expected that these factors do not predict treatment outcome.

Higher levels of pain and disability are known to predict persistence of pain and disability [25-27]. Both factors are typically not a reason to exclude patients from multidisciplinary treatment. In addition, reduction of symptoms is not the focus of a multidisciplinary pain treatment, in contrast to disability and interference of pain in daily life [6]. It is therefore expected that a low level of symptoms predicts a better treatment outcome. On the other hand because treatment is focused on the reduction of disability and the interference of pain with daily life, it is expected that disability looses its predictive value.

Finally, socio-demographic factors may predict the outcome of multidisciplinary treatment. Few studies [11] have focused on socio-demographic factors so far, thus it seems worthwhile to evaluate their predictive value.

In summary, depression, anxiety, illness beliefs, self-efficacy beliefs, the extent of symptoms, and socio-demographic factors are expected to predict the outcome of multidisciplinary treatment; whereas factors related to fear avoidance and disability are not. This study aims to evaluate these hypotheses on predictors of the outcome of multidisciplinary treatment in patients with CWP.

## Methods

### Design

The data of the present study were obtained in a longitudinal observational study. The baseline measurements (predictors and outcome measures) took place before start of treatment (T0). The second assessment (outcome measures) was performed 6 months after baseline [T1]. The ethical review board of Reade in Amsterdam approved this study. Written informed consent was obtained from each subject.

### Patients and procedure

Patients with CWP were referred by rheumatologists and general practitioners to the pain management team of our centre. Inclusion criteria for the study were: (i) a diagnosis of CWP according to the American College of Rheumatology criteria (ACR) [3]; (ii) eligible for multidisciplinary treatment according to the criteria the Dutch Consensus Report of Pain Rehabilitation [28], as assessed by both a rehabilitation physician and a psychologist; these criteria require patients to experience restrictions in daily living (e.g. sport, work) and/or psychosocial functioning; and (iii) age between 18 and 75 years. Exclusion criteria were: (i) pain resulting from known specific pathology; (ii) not eligible for multidisciplinary pain treatment because of a somatic disorder, social problem and/or psychiatric disorder (e.g. major depression), or because the patient was currently involved in a legal procedure of conflicting interest, was currently receiving pain treatment elsewhere, or was judged by the rehabilitation physician and/or psychologist not to be motivated for behavioural change; (iii) insufficient control of the Dutch language to complete questionnaires; and (iv) refusal to give informed consent.

A consecutive series of patients was included in the study. Recruitment took 14 months. The baseline measurements were integrated in the existing intake for the multidisciplinary treatment in our centre. For the follow-up measurements participants were phoned and letters were send to encourage them to return questionnaires.

### Intervention

The main goal of the intervention was to teach patients to cope with pain and to reduce the interference of pain in daily living. The treatment included cognitive behavioural therapy (CBT), the acquisition of pain management skills (e.g. goal setting, structuring of daily activities, pacing strategies, ergonomics), physical training (e.g. exercise), relaxation training, education about neuro-physiology and medication management, and assertiveness training. The components of the multidisciplinary treatment were in line with the core elements of multidisciplinary treatment in CWP [6,8]. In the treatment program ‘illness beliefs and self- efficacy beliefs’ were not explicit or standardized altered. The treatment was tailored to the patients personal goals and was performed in groups and on individual basis. Group treatment consisted of seven consecutive weeks of treatment, seven hours a week and was divided into two sessions a week of multidisciplinary treatment. Individual treatment was offered during a period of four to six months with a variable frequency per patient. The multidisciplinary team involved rehabilitation physicians, physiotherapists, occupational therapists, psychologists, and social workers. The multidisciplinary team discussed the treatment progress of patients during regular team meetings.

### Outcome measurements

Four outcome domains were defined in accordance with the recommendations of the Initiative on Methods, Measurement, and Pain Assessment in Clinical Trials (IMMPACT), i.e. pain, physical functioning, emotional functioning, and global perceived effect [29].

*Pain* was assessed with the Numerical Rating Scale Pain (NRS) (range 0–10). The endpoints of the scale are no pain and worst possible pain. Adequate psychometric properties have been documented [30].

*Physical functioning* was assessed with the Multidimensional Pain Inventory (MPI), subscale interference. The MPI consists of 13 empirically derived scales that measure different pain-related aspects. The subscale interference assesses patients perceptions about how pain interferes with their daily lives. A higher score of interference (range 0 to 54) means more interference of pain in daily life. The MPI has been widely used with diverse chronic pain samples and has good psychometric properties [31].

*Emotional functioning* was assessed with the Beck Depression Inventory II (BDI-II). The BDI-II is a self-reported measure that assesses the cognitive, affective, and somatic symptoms of depression [32,33]. The 21 questions ask about symptoms and attitudes and are rated from 0 to 3 in terms of intensity. A higher score on the BDI-II (range 0 to 63) indicates more depressive symptoms. The BDI-II has been shown to have adequate psychometric properties [34].

*Global perceived effect* (GPE) due to treatment was assessed with a 7-point Likert scale ranging from the complaints in global health ‘have disappeared’ to ‘very much deteriorated’. The scale was dichotomized in 0 = no change/worse and 1 = improved.

### Potential predictors of treatment outcome

#### Psychological distress

*Depression* was assessed with the BDI-II. The BDI-II has been described previously.

*Psychological functioning* was assessed with the Dutch version of the Symptom CheckList (SCL90). The SCL 90 is a self reported questionnaire and measures psychological and physical distress. The items are rated on a 5 point scale: 1 (not at all) to 5 (very much). The total score of the SCL 90 (range 90 to 450) reflects psychoneurotism, where a higher score means a higher degree of psychoneuroticism. The Dutch version of the SCL-90 has been shown to have adequate psychometric properties [35].

*Anxiety* was assessed with the Hospital Anxiety Depression Scale (HADS), subscale anxiety. The scale measures anxiety based on 7 items. A higher score (range 0 to 21) means a higher level of anxiety. The HADS is widely used and have adequate psychometric properties [36].

#### Illness and self-efficacy beliefs

*Illness beliefs* were assessed with the Revised Illness Perceptions Questionnaire (IPQ-R). Seven subscales of the Revised Illness Perceptions Questionnaire (IPQ-R) were used to measure beliefs about *1)‘consequences’-* expected effects and outcome of the illness*; 2)‘coherence’-* patient’s logical and complete understanding of the illness*; 3)‘emotional representations’-* negative emotional reactions like anger and fear related to the illness; *4) ‘timeline’-* chronic timeline expectancies of the illness; *5) ’timeline cyclical’-* expectancies on the variability of the illness; *6)‘personal control’-* extent to which patients believe they can control the illness; *and 7) ‘treatment control’*- belief in treatment and recommended advice. Items in these scales are rated on a 5-point Likert scale ranging from ’strongly disagree’ to ‘strongly agree’. Higher scores on personal control (range 6 to 30), treatment control (range 5 to 25) and low scores on illness coherence (range 5 to 25) demonstrate positive beliefs about the controllability of the illness and a personal understanding of the illness, respectively. High scores on timeline (range 6 to 30), timeline cyclical (range 4 to 20), consequences (range 6 to 30) and emotional representation (range 6 to 30) demonstrate strongly held beliefs about the chronicity of the illness, the cyclical nature of the illness, negative consequences of the illness, and more emotional representations, respectively [37,38]. The validity and reliability of the IPQ-R have been documented [39].

*Self-efficacy* beliefs were measured with the Dutch General Self-efficacy Scale (DGSS). General self-efficacy is defined as a broad and stable sense of personal competence to deal effectively with a variety of stressful situations [40]. The DGSS consists of 10 items, and are answered on a four point scale, ranging from ‘not at all true’ to ‘exactly true’, with a higher score (range 10 to 40) indicating a higher self-efficacy. The psychometric properties of the DGSS have been documented [41,42].

#### Fear-avoidance beliefs and behaviour

*Fear-avoidance* was measured with the Tampa Scale of Kinesiophobia (TSK). The TSK measures self-reported fear of movement and (re) injury. The scale consists of 17 items and the items are evaluated on a 4-point Likert scale, ranging from ‘strongly disagree’ to ‘strongly agree’. A higher score (range 17 to 68) indicates a higher degree of fear-avoidance [43].

*Avoidance behaviour* was measured with the Pain Coping Inventory (PCI), subscale resting. The PCI subscale resting assesses the level to which patients avoid physical activity. The scale consists of five items, and is rated on a 4-point Likert scale ranging from ‘rarely or never, to very frequently’. A higher score (range 5 to 20) on the PCI means more avoidance. Adequate psychometric properties have been documented [44].

*Catastrophizing* was measured with the Dutch adaptation of the Coping Strategy Questionnaire (CSQ), subscale catastrophizing. The subject indicated to what extent this particular coping strategy was utilized, based on 6 items. A higher score indicated (range 0 to 60) that the subject made more use of this coping style. The validity and reliability of the CSQ have been documented [45].

#### Symptoms

*Pain* was assessed with the NRS. The NRS has been described previously (see above).

*Impact of FM* was assessed with the Fibromyalgia Impact Scale (FIQ). The scale evaluates physical functioning, work status, depression, anxiety, sleep, pain, stiffness, fatigue, and well-being. A higher score (range 0 to 80) indicate a higher impact of FM. The FIQ has been widely used with diverse chronic pain samples, and has good psychometric properties [46].

*Fatigue* was measured with the subscale fatigue of the FIQ. A higher score indicate a higher level of fatigue.

#### Physical functioning

*Physical functioning* was assessed with the MPI -subscale interference. The MPI has been described previously (see above).

*General activity* was measured with the MPI -subscale general activity. The subscale general activity (range 0 to 18) assesses the frequency of activity of household chores, outdoor work, activities away from home, and social activities.

#### Socio-demographic variables

*Socio-demographic variables* were recorded of each patient: age was rated in years and gender, partnership, ethnicity and level of education were coded as dummy variables.

### Statistical analyses

Descriptive statistics for baseline patient characteristics were tabulated as means (SD) or medians (IQR) if data was not normally distributed. Patient characteristics (i.e. age, pain, interference of pain and depression) of participants with complete datasets (N=120) and drop-outs (N=13) were compared and analyzed with a Mann–Whitney *U* test. Differences between T0 and T1 scores on the outcome measures BDI-II, MPI- subscale interference and NRS pain were analyzed using two tailed paired t–tests (with a significance level of *P* < 0.05). Univariate and multiple regression analyses were performed to evaluate the predictors of treatment outcome. The change in the outcome variables and GPE were entered as the dependent variable. The potential predictors were entered as independent variables. First an explorative univariate regression analysis was performed with each potential predictor of the outcome of multidisciplinary treatment. Variables that reached a statistical significance below 0.20 were selected as potential predictors for the multivariate analyses. The bivariate correlation coefficients were computed to establish the relationships between the selected predictors of the outcome variables that reached a statistical significance at *P* < 0.20. When correlations coefficients of r > 0.70 were found, one of the variables was excluded from the model, to avoid multicolinearity. Multiple linear regression analyses (backward selection) were performed to identify predictors of change in outcome on NRS pain, MPI- subscale interference and BDI-II. A multiple logistic regression analysis (backward selection) was performed to identify predictors of GPE. A significance level of *P* < 0.05 was used to include a predictor in the final model. In secondary analyses, the variable ‘treatment frequency’ was forced into the final model of the multiple regression analysis to determine whether treatment frequency was a potential confounder of the found relationships.

## Results

### Study population

Of the 361 patients referred to and evaluated by the pain management team, 165 patients fulfilled the inclusion criteria for this study. Of these patients, 138 consented to participate in the study. Reasons for not participating were: dyslexia, not interested in completing the necessary paperwork, too busy or no specific reason. Of these 138 participants, 133 participants provided data at T0. Reasons for missing cases at this stage were: withdrawal from treatment (n=3) and no specific reason (n=2). One hundred twenty participants provided data at T1. Reasons for missing cases at this stage were: no further consent for participation in the study (n=3) or no specific reason (n=10). Participants who dropped-out of the study at T1 did not differ significantly from participants who completed the measurements at T1, except for depression (M_drop-out_ = 26.23, SD = 7.36; M_participants_ = 20.78, SD = 8.85; *P =* 0.02). Baseline characteristics of the participants are shown in Table 1.

**Table 1:** Baseline patient characteristics

**Variable**	**Value**	**Range**	**N**
Pain (NRS), mean (SD)	6.1 (2.1)	1-10	120
Interference (MPI), mean (SD)	4.1 (1.1)	1.1-5.9	120
Depression (BDI-II), mean (SD)	20.8 (8.9)	4 –51	120
Psychological functioning (SCL 90), mean (SD)	179.3 (48.6)	108-409	116
Anxiety (HADS), mean (SD)	8.1 (3.6)	0-17	116
Emotional representation (IPQ), mean (SD)	19.2 (4.7)	8-30	120
Coherence (IPQ), mean (SD)	15.0 (4.8)	6-25	119
Consequence (IPQ), mean (SD)	20.9 (4.4)	10-30	120
Personal control (IPQ), mean (SD)	18.6 (4.3)	6-30	120
Treatment control (IPQ), mean (SD)	16.2 (2.9)	5-25	120
Timeline cyclical (IPQ ), mean (SD)	15.3 (3.2)	4-20	120
Timeline (IPQ), mean (SD)	23.6 (3.6)	12-30	120
General self efficacy (DGSS), mean (SD)	2.9 (.6)	1.2- 4.1	120
Fear avoidance (TSK), mean (SD)	36.5 (7.5)	21-56	120
Avoidance behavior (PCI), mean (SD)	2.5 (.6)	1-4	120
Catastrophizing (CSQ), mean (SD)	23.9 (11.0)	1.0-50.0	120
Impact of FM (FIQ), mean (SD)	51.8 (11.0)	25.8 – 77.8	120
Fatigue (FIQ), median (IQR)	8.0 (7.25; 10.0)	4-10	120
Activity level (MPI), mean (SD)	2.6 (.9)	0-5.1	120
Gender, Female (%)	95		114
Age	45.0 (10.3)	21-69	120
Partnership, Yes (%)	51		n= 61
Ethnicity (%)			
Native	71		n= 85
Western non native	13		n= 15
Non western non native	17		n= 20
Education (%)			
Primary	18		n= 21
Secondary	50		n= 59
High	33		n= 39

Values are means (SD), median (IQR) or percentages. SD= standard deviation, IQR= interquartile range. BDI-II= Beck Depression Inventory, CSQ= Coping Scale Questionnaire, HADS= Hospital Anxiety Depression scale, DGSS= Dutch General Self efficacy Scale, FIQ= Fibromyalgia Impact Questionnaire, IPQ= Illness Perception Questionnaire, MPI= Multidimensional Pain Inventory, NRS= Numerical Rating Scale, PCI= Pain Coping inventory, SCL 90= Symptom checklist, TSK= Tampa scale for Kinesiofobia.

### Changes in depression, interference of pain, pain and global perceived effect

Table 2 shows T0 and T1 scores on the outcome measures. Statistically significant improvements after treatment were found for interference of pain (MPI) (*P=* 0.02) and depression (BDI-II) (*P<* 0.001). Forty-eight percent of the patients improved on GPE.

**Table 2:** Outcome measures before and after intervention

	**T0**	**T1**	***P *value**
Pain (NRS)	6.08 (2.08)	6.08 (1.89)	0.96
Interference (MPI)	4.07 (1.06)	3.87 (1.13)	0.02
Depression (BDI-II)	20.69 (8.83)	17.61 (9.52)	< 0.001
Perceived global effect			
No change/worse		51.7%	
Improved		48.3%	

Values are means (SD), or percentages, SD= standard deviation.

T0= before intervention, T1= follow up at 6 months.

### Predictors of outcome

The results of the univariate linear regression analyses are shown in Table 3. Predictors with a relationship with the outcome measure of *P* < 0.20 were used for multiple regression analyses. The correlation coefficients between the potential predictors of the outcome measure *pain* ranged from .27 (i.e. pain (NRS) and depression (BDI)) to .52 (i.e. pain (NRS) and the impact of fibromyalgia (FIQ)). The relationships between the potential predictors of the outcome measure *interference of pain* ranged from .18 (self-efficacy (DGSS) and beliefs in consequences (IPQ)) to .67 (i.e. psychological functioning (SCL 90) and anxiety (HADS)). In addition, the relationships between the potential predictors of the outcome measure *depression* ranged from .21 (i.e. beliefs in timeline (IPQ) and resting (PCI)) to -.31 (i.e. beliefs in personal control (IPQ) and resting (PCI)). Finally, relationships between the potential predictors of the outcome measure *global perceived effect* ranged from -.20 (i.e. timeline beliefs (IPQ) and treatment control (IPQ)) to .62 (i.e. fatigue (FIQ) and impact of fibromyalgia (FIQ)). Only variables that showed a statistically significant association (at *P* < 0.05) with change scores in the outcome measures were included in the final model. The results of the multiple linear regression analyses are shown in Table 4. *Greater improvement in pain (NRS)* was associated with more pain (NRS) at baseline (*P* < 0.001) and male gender (*P* = 0.02). *Greater improvement in interference of pain in daily life (MPI)* was associated with more interference of pain (MPI) at baseline (*P* < 0.001), less anxiety (HADS) (P = 0.03), stronger beliefs in personal control (IPQ) (*P* < 0.004), less beliefs in consequences (IPQ) (*P* < 0.001), male gender (*P* = 0.02), and a higher level of education (*P*= 0.003, *P* = 0.03). *Greater improvement in depression (BDI-II)* was associated with more depressive symptoms at baseline (*P* < 0.001), stronger beliefs in personal control (*P* < 0.001), and a higher level of education (*P* = 0.02, *P* = 0.04). *Better global perceived effect* was associated with less pain (NRS) (*P* = 0.01), less fatigue (FIQ) (*P* = 0.03), and a higher level of education (*P* = 0.03). The explained variance (R^2^) for the final models ranged from 25.7% to 38.1%. In summary, five of our eight hypotheses were confirmed while one hypothesis was partly confirmed (see Table 5).

**Table 3:** Results of univariate regression analyses of change in pain, interference of pain, depression and GPE

	**NRS Pain**			**MPI Interference**			**BDI- II Depression**			**GPE**	
	**B**	**β**	***P***	**B**	**β**	***P***	**B**	**β**	***P***	**Odds**	***P***
Pain (NRS)	-.53	-.56	**.00**	.04	.10	.27	.05	.01	.89	.70	**.00**
Interference (MPI)	-.12	-.06	.49	-.26	-.32	**.00**	.04	.01	.95	.70	**.05**
Depression (BDI-II)	-.04	-.17	**.06**	.00	.04	.69	-.28	-.33	.**00**	1.00	.84
Psychological functioning (SCL 90)	-.00	-.07	.45	.00	.17	**.06**	-.02	-.12	.21	1.00	.98
Anxiety (HADS)	-.06	-.10	.27	.04	.17	**.07**	-.24	-.11	.23	1.06	.24
Emotional representation (IPQ)	.01	.01	.88	.01	.06	.52	-.15	-.10	.31	.98	.65
Coherence (IPQ)	.04	.10	.30	.02	.13	**.15**	.14	.09	.33	.10	.90
Consequences (IPQ)	-.04	-.08	.39	.03	.15	**.10**	-.01	-.00	.97	.96	.41
Personal control (IPQ)	-.02	-.04	.70	-.03	-.16	**.09**	-.51	-.30	**.00**	1.14	**.01**
Treatment control (IPQ)	-.06	-.09	.35	-.19	-.08	.42	-.19	-.08	.42	1.15	**.04**
Timeline cyclical (IPQ)	.05	.08	.37	-.02	-.06	.51	.07	.03	.75	1.18	**.01**
Timeline (IPQ)	-.00	-.01	.93	.03	.12	**.20**	.31	.15	**.11**	.97	.54
General self efficacy (DGSS)	.36	.11	.23	-.27	-.19	**.04**	.99	.08	.41	.97	.93
Fear avoidance (TSK)	-.03	-.10	.29	.00	.02	.85	.07	.07	.45	1.02	.52
Avoidance behaviour (PCI)	.07	.02	.81	.02	.02	.87	1.91	.16	**.09**	.90	.71
Catastrophizing (CSQ)	-.02	-.10	.30	0.00	-.00	.99	-.03	-.04	.67	1.00	.81
Impact of FM (FIQ)	-.04	-,21	**.02**	.01	.10	.27	-.00	-.00	.98	.94	**.00**
Fatigue (FIQ)	-.05	-.04	.65	.05	.08	.39	.08	.02	.87	.68	**.00**
Activity level (MPI)	-.10	-.04	.65	-.04	-.04	.67	.47	.05	.57	1.46	**.10**
Gender	1.40	.16	**.09**	.60	.15	**.10**	3.25	.10	.30	.17	**.12**
Age	-.03	-.17	**.07**	-.02	-.20	**.03**	.06	.09	.34	.98	.30
Partnership	-.18	-.05	.61	.05	.03	.75	-.41	-.03	.77	1.07	.85
Ethnicity											
Native vs Western non native	-.20	-.04	.71	-.19	-.08	.43	−2.97	-.13	**.16**	.59	.36
Native vs non Western non native	-.32	-.06	.51	.02	.01	.94	1.53	.08	.41	.48	**.15**
Education											
Primary vs secondary	.09	.02	.87	-.50	-.29	.**02**	−4.69	-.31	**.02**	2.96	.**05**
Primary vs high	-.18	-.04	.74	-.39	-.22	**.09**	−5.71	-.36	**.01**	2.38	.14

B = unstandardized regression coefficient, β = standardized regression coefficient, *P* = *P* value, in bold: *P* values ≤ 0.20. BDI-II= Beck Depression Inventory, CSQ= Coping Scale Questionnaire, DGSS= Dutch General Self efficacy Scale, FIQ= Fibromyalgia Impact Questionnaire, GPE= Global perceived effect, IPQ= Illness Perception Questionnaire, MPI= Multidimensional Pain Inventory, NRS= Numerical Rating Scale, PCI= Pain Coping inventory, SCL 90= Symptom checklist, TSK= Tampa scale for Kinesiofobia.

**Table 4:** Results of multiple regression analyses of change in pain, interference of pain, depression and GPE

	**NRS Pain**			**MPI Interference of pain**			**BDI II Depression**			**Global perceived effect**	
	**N = 120**			**N=113**			**N = 116**			**N = 119**	
	***B *(95% CI)**	**β**	***P***	***B *(95% CI)**	**β**	***P***	***B *(95% CI)**	**β**	***P***	**Odds (95% CI)**	***P***
Baseline value	-.53 (−.67 to -.39)	-.57	<0.001	-.55 (−.70 to -.39)	-.68	<0.001	-.30 (−.44 to -.16)	-.35	<0.001		
Anxiety (HADS)				.043 (.00 to .08)	.18	0.03					
Personal control (IPQ)				-.05 (−.08 to-.02)	-.25	0.004	-.53 (−.82 to -.23)	-.30	<0.001		
Consequence (IPQ)				.08 (.04 to .11)	.40	<0.001					
Pain (NRS)										.75 (.60 to .93)	0.01
Fatigue (FIQ)										.72 (.53 to .96)	0.03
Gender	1.54 (.22 to 2.86)	.17	0.02	.74 (.11 to 1.38)	.18	0.02					
Education											
Primary vs secondary				-.57 (−.93 to -.20)	-.34	0.003	−4.09 (−7,57 to -.62)	-.27	0.02	3.83 (1.14 to 12.86)	0.03
Primary vs high				-.43 (−.82 to -.04)	-.24	0.03	−3.93 (−7.71 to -.15)	-.25	0.04	2.52 (.73 to 8.67)	0.14
*R*^*2*^	34.6%			38.1%			25.7%				

*B* = unstandardized regression coefficient, 95% CI = 95% confidence interval of *B*, β = standardized regression coefficient, *P* = *P* value global perceived effect: 0 = no change/worse, 1 = improved, gender; 0 = male, 1= female. β negative: greater improvement in BDI- II, NRS pain and MPI interference. β positive: less improvement in BDI- II, NRS pain and MPI interference. Higher scores on the HADS, IPQ, NRS pain and FIQ, indicate more anxiety, higher beliefs in personal control, higher beliefs in consequence, more pain and fatigue.

**Table 5:** Summary of hypothesis testing

**Hypotheses**	**Confirmed**
Depression predicts a poor outcome of multidisciplinary treatment	No
Anxiety predicts a poor outcome of multidisciplinary treatment	Yes
Negative illness beliefs predict a poor outcome of multidisciplinary treatment	Yes
Low self-efficacy beliefs predict a poor outcome of multidisciplinary treatment	No
High level of symptoms predict a poor outcome of multidisciplinary treatment	Yes
Demographic factors (age, gender, partnership, ethnicity and education) predict the outcome of multidisciplinary treatment	Partly (i.e. gender and education)
Fear avoidance beliefs and behaviour do not predict the outcome of multidisciplinary treatment	Yes
Level of disability does not predict the outcome of multidisciplinary treatment	Yes

In the secondary analyses, adjustment for treatment frequency in the multiple variable models only affected the model of GPE. After adjustment, global perceived effect was only associated with pain (NRS) (OR = .72; 95% CI, .58 to 90), fatigue and education were no longer factors in the final model.

## Discussion

The present results show that psychological distress (i.e. anxiety), illness beliefs (i.e. beliefs in personal control and consequences), symptoms of pain and fatigue, and socio-demographic factors (i.e. gender and level of education) are associated with the success of multidisciplinary treatment in patients with CWP. In addition, for all outcome measures, higher baseline values (i.e. indicating a worse health status) were associated with greater improvement in outcome.

In line with one of our hypotheses, we found that higher levels of anxiety at baseline were associated with less improvement in interference of pain with daily life after multidisciplinary treatment. To our knowledge, this is the first study that has focused on anxiety as a potential predictor of multidisciplinary treatment outcome in patients with CWP. These results indicate that higher levels of anxiety are a barrier to effective multidisciplinary treatment. Contrary to our hypothesis derived from the literature [11] that depression would be a predictor of treatment outcome, we found no evidence to support this. A selective dropout from the study of patients with more depressive symptoms might explain this. In addition, exclusion of severely depressed patients might have contributed to depression failing to predict outcome in the present study. Alternatively, although depression was not specifically targeted during treatment, the level of depression may have been influenced by various components of the pain management program (e.g. CBT, goal setting and structuring of daily life) and therefore may have lost its predictive value.

As expected, stronger beliefs in personal control were associated with greater improvement of depression and interference of pain with daily life after multidisciplinary treatment. In addition, less belief in the negative consequences of the illness appeared to be associated with a greater improvement in interference of pain with daily life. These results are congruent with those of Glattacker et al. [47], who demonstrated that positive illness beliefs (e.g. less belief in consequences) are associated with a greater improvement in treatment outcome in women with fibromyalgia. Contrary to our expectations and our preliminary evidence [11], we did not find self-efficacy to predict treatment outcome. This might be explained by the operationalization of the concept of self-efficacy. In this study we were interested in the predictive value of general self-efficacy beliefs (i.e. defined as a broad and stable sense of personal competence to deal effectively with a variety of stressful situations). We did not measure domain-specific self-efficacy. It is possible that general self-efficacy at baseline is not directly related to treatment outcome, while this might be true for domain-specific self-efficacy beliefs [48].

Fear-avoidance beliefs and behaviour did not predict the outcome of multidisciplinary treatment. We expected that fear-avoidance beliefs and behaviour would not predict treatment outcome because they are often targeted during multidisciplinary treatment to increase daily activity and therefore lose their predictive value.

As expected, patients with lower levels of pre-treatment pain and fatigue reported a better global treatment effect. This suggests that patients with pronounced symptoms benefit less from standard multidisciplinary treatment including CBT. Thieme et al. [49] also found that patients with pronounced pain benefit less from multidisciplinary treatment which includes CBT. Better results have been found with specific operant behaviour therapy (OBT) treatments which focus specifically on the modification of pain behaviour: pain behaviour is not endorsed or rewarded. As expected, physical functioning was not a predictor of treatment outcome. This can be explained through the fact that the focus of multidisciplinary treatment is on the reduction of disability and interference of pain with daily life, whereby physical functioning looses its predictive value.

Finally, the results indicate that socio-demographic factors predict the outcome of multidisciplinary treatment. Male gender predicts a greater improvement in interference of pain with daily life and post treatment pain. Since our study population only included 5 men, this finding requires further investigation. In addition, a higher level of education predicts a greater improvement in depression, interference of pain with daily life and a better global perceived effect. These results are in line with the findings of other studies in FM patients [50,51]. Our findings might be explained by low health literacy: low educational status is correlated with low health literacy in patients with chronic diseases [52,53]. In accordance with our clinical experience, patients with a lower level of education may have difficulty in fully understanding the presented material and thus applying it in practice.

Another finding was that higher baseline values of outcome measurements (indicating more depressive symptoms, higher interference of pain with daily life, and more pain) were associated with greater effects of multidisciplinary treatment. This can be explained by a methodological effect (i.e. regression to the mean) [54]: patients with high baseline scores can show more improvement than patients with already low baseline scores.

There are several limitations in our study that warrant discussion. We chose our predictors based on theory and previous research instead of an explorative approach. However, this still led to a relatively large number of predictors which we reduced using statistical pre-selection. This procedure may have caused some instability in our results [55]. Furthermore, we did not include a control group and are thus not able to conclude that changes in outcome measurements were the result of the multidisciplinary treatment. Evaluating predictors of change in uncontrolled studies does not make it possible to distinguish between predictors of natural course of a disease and predictors of successful treatment. Whether or not a distinction in predictors exists can only be studied in randomized controlled trials aiming at investigating the effectiveness of multidisciplinary treatment in patients with CWP.

Although the improvements in outcome were small on a group level, individual differences in patients characteristics were related to the differences in the effect of treatment outcome. For multidisciplinary treatment this suggests that it is important to find out which clinical characteristics are present in each patient with CWP. Tailoring treatment or strategies to specific patient characteristics may further improve multidisciplinary treatment outcome.

The present results may have implications for treatment planning. Patients with pronounced psychological distress (i.e. anxiety), or negative illness beliefs might benefit from treatment components which specifically focus on the treatment of psychological distress or maladaptive thoughts about their illness, respectively. For these patients it might be worthwhile to add these specific components to multidisciplinary treatment. In addition, as pronounced symptoms predicted poorer treatment outcome, an approach based on operant behaviour therapy may be more effective than an approach based on cognitive behaviour therapy [49]. With regard to the finding that education predicted treatment outcome, it might be worthwhile to adjust multidisciplinary treatment to the cognitive ability of the patient. Alternatively, when there is no option to adapt the treatment program to the patients personal needs, selection of eligible patients (i.e. patients with less anxiety, more positive illness beliefs, less pronounced symptoms and/or a higher educational level) might also improve the effectiveness of a multidisciplinary treatment. Finally, little is known about the influence of treatment intensity on treatment response. Adjustment of treatment frequency of the results did not influence the relationships between predictors and outcome measurements, with the exception of GPE. Additional studies are necessary to develop a greater understanding of the influence of treatment intensity on predictors and multidisciplinary treatment outcome in patients with CWP.

## Conclusion

In conclusion, less anxiety, stronger beliefs in personal control, lower beliefs in consequences, less pain, less fatigue and a higher level of education are predictors of greater improvement in the outcome of multidisciplinary treatment. Tailoring treatment to specific patient characteristics or selecting eligible patients for multidisciplinary treatment may further improve treatment outcome.

## Competing interests

The authors declare that they have no competing interests.

## Authors’ contributions

JD, MS and AR designed the study. AR coordinated the data collection, performed the statistical analysis. AR, JD and MvdL wrote the manuscript. MS helped to draft the manuscript. LR participated in the design of the study and helped to draft the manuscript. All authors read and approved the final manuscript.

## Pre-publication history

The pre-publication history for this paper can be accessed here:

http://www.biomedcentral.com/1471-2474/14/133/prepub
